# Biocompatible Fe-Based Micropore Metal-Organic Frameworks as Sustained-Release Anticancer Drug Carriers

**DOI:** 10.3390/molecules23102490

**Published:** 2018-09-28

**Authors:** Xin Leng, Xiaoxv Dong, Wenping Wang, Na Sai, Chunjing Yang, Longtai You, Hongliang Huang, Xingbin Yin, Jian Ni

**Affiliations:** 1 School of Chinese Material Medical, Beijing University of Chinese Medicine, Beijing 102488, China; 20160931927@bucm.edu.cn (X.L.); wangwenp6@163.com (W.W.); yxsaina@126.com (N.S.); ylt_svip@163.com (L.Y.); 20160941191@bucm.edu.cn (C.Y.); 2 Beijing Research Institute of Chinese Medicine, Beijing University of Chinese Medicine, Beijing 100029, China; dxiaoxv@163.com; 3 School of Pharmacy, Inner Mongolia Medical University, Hohhot 010110, China; 4 National Center for International Joint Research on Membrane Science and Technology, Tianjin Polytechnic University, Tianjin 300387, China; 5 State Key Laboratory of Separation Membranes and Membrane Processes, Tianjin Polytechnic University, Tianjin 300387, China

**Keywords:** MIL-53(Fe), oridonin, sustained-release, antitumor

## Abstract

Sustained-release preparation is a hot spot in antitumor drug research, where the first task is to select suitable drug carriers. Research has revealed that carboxylic acid iron metal–organic frameworks (MOFs), constructed from iron (Fe) ions and terephthalic acid, are nontoxic and biocompatible. Due to the breathing effect, the skeleton of this mesoporous material is flexible and can reversibly adapt its pore size through drug adsorption. Therefore, we chose one kind of Fe-MOF, MIL-53(Fe), as a carrier for the anticancer drug oridonin (Ori). In this work, we report the design and synthesis of MIL-53(Fe) and explore its ability as a transport vehicle to deliver Ori. MIL-53(Fe) is characterized by scanning electron microscopy and X-ray powder diffraction. A loading capacity of 56.25 wt % was measured by high performance liquid chromatography. This carrier was safe and nontoxic (cell viability > 95.27%), depending on the results of 3-(4,5-dimethylthiazol-2-yl)--2,5-diphenyltetrazolium bromide assays, lactate dehydrogenase assays, and Annexin V-fluoresce isothiocyanate/propidium iodide double-staining assays. After loading the drug, the structure of the MIL-53(Fe) was not destroyed, and Ori was amorphous in MIL-53(Fe). Based on an analysis of the Ori release profile, results suggest that it lasts for more than seven days in vitro. The cumulative release rate of Ori at the seventh day was about 82.23% and 91.75% in phosphate buffer saline solution at 37 °C under pH 7.2 and pH 5.5, respectively. HepG2 cells were chosen to study the cytotoxicity of Ori@MIL-53(Fe), and the results show that the anticancer ratio of Ori@MIL-53(Fe) system reaches 90.62%. Thus, MIL-53 can be used as a carrier for anticancer drugs and Ori@MIL-53(Fe) is a promising sustained-release drug delivery system for the cancer therapy.

## 1. Introduction

Malignant tumors are a kind of disease that seriously threaten the length and quality of human life. The second highest mortality rate in humans among all kinds of diseases is due to malignant tumors. Liver cancer is the third most common cancer [1]. The treatment of liver cancer primarily involves high doses of chemotherapy and surgical treatment. New chemotherapeutic drugs and drug delivery systems (DDS) are urgently needed to enhance the curative effect or to reduce the side effects of cancer treatment.

**Figure 1:**
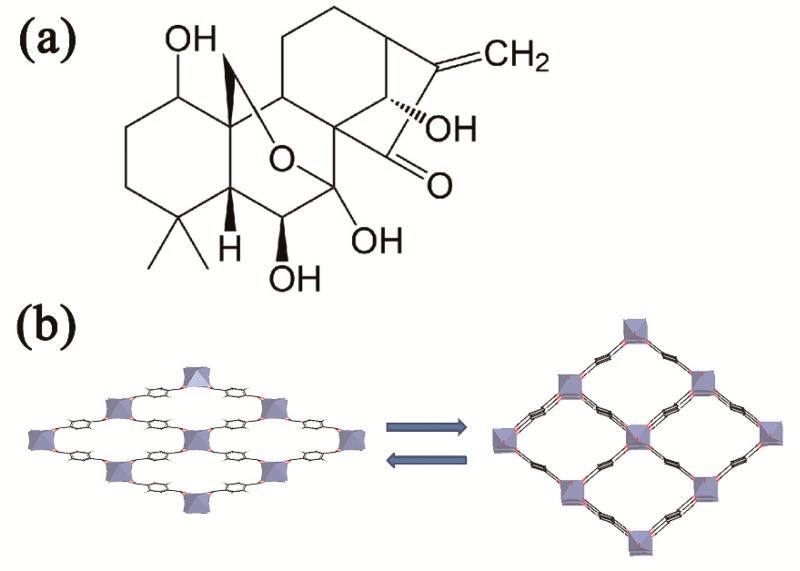
Structural Diagram. (**a**) chemical Structure of Ori and (**b**) respiratory effect on the mutual conversion of large and small pore of MIL-53(Fe).

Oridonin (Ori) is an *ent*-kaurene diterpenoid compound (Figure 1a), extracted from the medicinal herb *Rabdosia rubescenes* [2]. Past studies found that oridonin has conspicuous anti-tumor activity in many types of human cancer, such as triple-negative breast cancer cells [3,4], osteosarcoma cells [5], human hepatoma cells [6], prostate cancer cells [7], and myeloma cells [8]. Therefore, it has attracted wide attention. A study of the mechanism of Ori’s anti-hepatocellular activity showed that it reduces G2/M arrest and apoptosis in human liver tumor cells [9] and induces HepG2 cell apoptosis by oxidative stress pathways. Prdx2, Hsp70-1, and are involved in the anticancer activity of Ori [10]. Despite the strong anticancer activity of Ori, its poor solubility in water or common oil, moderate hydrophobicity, chemical instability, and short biological half-life, all limit its clinical use. Moreover, when it is dissolved, the light and heat may lead to active group (α-methylene-cyclopentanone) destruction, further limiting its practical applicability [11,12]. Therefore, finding a new drug carrier to increase the stability of oridonin and improve its in vivo efficiency is important for expanding its clinical anti-tumor applications.

Two kinds of drug delivery systems have been widely recognized: organic systems, including polymers [13,14], liposomes [15], micelles [16,17], and protein nanoparticles [11]; and inorganic systems, such as mesoporous silica nanoparticles [18,19,20], graphene [21], and zeolites [22]. Although organic carriers are biocompatible, the disadvantages of low loading and instability are difficult to overcome [23]. Inorganic carriers have well-defined porosity, stability, high drug loading, and enable controlled drug release, but they are hard to degrade and be removed from body [24]. Based on the above research, metal–organic frameworks (MOFs) are being widely used as drug carriers due to their unique characteristics, for example, high porosity, large specific surface area, surface paintable, pore size tunable, structural stability, non-toxicity, and biocompatibility [25,26,27,28], The Materials of Institut Lavoisiser (MIL) series is a famous type of MOF, characterized by the extremely flexible skeleton of the material, and the material structure changes between having a big hole and a narrow hole when stimulated. MIL-53(Fe) is an iron (III) carboxylate MOF [29,30]. Parallel trans corner-sharing iron (III) octahedral chains, each of which are cross-linked by 1,4-benzendicarboxylate (BDC) anions, form a one-dimensional lozenge-shaped pore channel system, which constitute the structure of MIL-53(Fe) [31]. MIL-53(Fe) can open its pores in the presence of guest drugs; hence, the skeleton of this microporous material flexibly adsorbs drugs, as shown in Figure 1b. The characteristic of MIL-53(Fe) is called “breathing” [32,33]. In this work, we used MIL-53(Fe) to absorb the Ori using the solvent diffusion technique. The corresponding drug-loaded MIL-53(Fe) is named Ori@MIL-53(Fe). The calculated Ori-loading capacity reached up to 56.25 wt % through optimizing the best loading process. The Ori@MIL-53(Fe) was evaluated for release profiles at 37 °C in pH 5.5 and pH 7.2 phosphate buffer saline (PBS) solution. Ori was more slowly released, which further improved the anti-liver cancer activity. In addition, in vitro tests showed that MIL-53(Fe) had a good chemical stability with non-cytotoxicity.

## 2. Results

### 2.1. Preparation and Characteristion of MIL-53(Fe)

**Figure 2:**
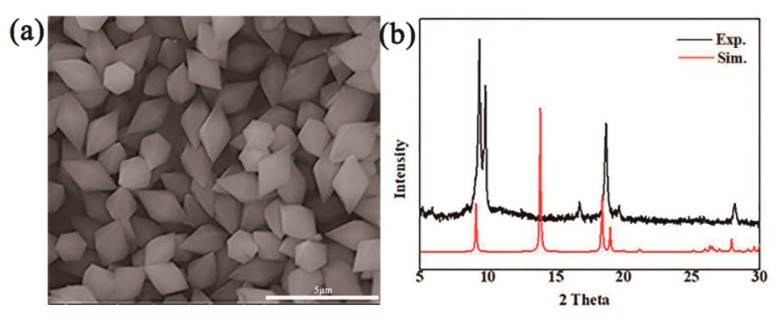
Characteristics of MIL-53(Fe). (**a**) SEM image of MIL-53(Fe), which is octahedral in diamond-shape, owing to corner-sharing chains of iron ion clusters connected through terephthalate linkers, and channels size about 2-3μm. (**b**) XPRD spectra of MIL-53(Fe).

MIL-53(Fe) can adapt its porosity and optimize drug-matrix interactions given its flexible framework, which allows it to maximize combination interactions and minimizing steric hindrance [31]. The method of synthesizing MIL-53(Fe) is simple and inexpensive. Furthermore, MIL-53(Fe) is chemically stable and has low toxicity, and the trace amount of dissociative iron ions can benefit for the human body.30 The prepared MIL-53(Fe) was rhombohedral, as shown in the scanning electron microscopy (SEM) images, which indicated that MIL-53(Fe) had a diameter of approximately of 2.5–3.0 μm (Figure 2a).

**Figure 3:**
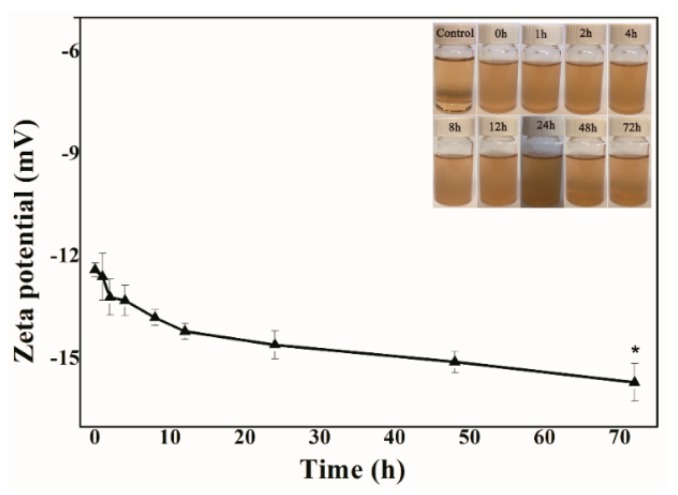
Zeta potential curves with time of MIL-53(Fe) in media serum at 37 °C.

X-ray powder diffraction (X-PRD) analysis was performed to analyze the powder purity of MIL-53(Fe) at room temperature (Figure 2b), and the main crystalline peaks were obvious at 9.12°, 18.05°, 19.25°, and 28.14°, which agreed well with the simulated peak pattern. The Zeta potential of nanoparticle surface severely influences the dispersion stability of nanoparticles. Therefore, the Zeta potential also reflects the stability of the dispersion system. Figure 3 shows the change curve of the Zeta potential with time when MIL-53(Fe) was dispersed in fetal bovine serum (FBS). MIL-53(Fe) has good dispersion and stability serum media at 37 °C within 48 h.

### 2.2. Optimal Loading Process and Characterization of Ori@MIL-53(Fe)

**Figure 4:**
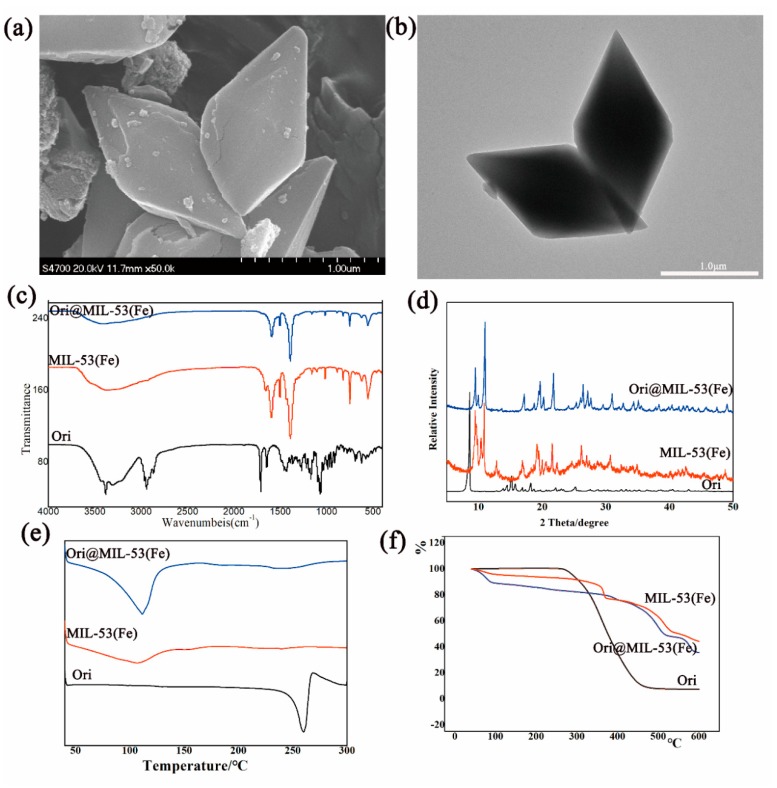
Characteristic of Ori@MIL-53(Fe): (**a**) SEM, (**b**) TEM, (**c**) FTIR, (**d**) XPRD, (**e**) DSC and (**f**) TGA.

By using the L_9_(3^4^) orthogonal table, three factors: proportion of Ori and MIL-53(Fe) (A), mixing time (B), and the amount of solvent (C), were selected to be optimized. No significant difference was found in the three factors (*p* > 0.05). The drug-loading rate reached up to 56.25 wt % under optimized conditions: MIL-53(Fe): Ori (1:4), magnetic stirring for 4 days, and MIL-53(Fe): methanol (1:0.5). The size and characteristics of the Ori@MIL-53(Fe) are shown in Figure 4. 

Through the SEM (Figure 4a) and transmission electron microscope (TEM, Figure 4b) images of Ori@MIL-53(Fe), the MIL-53(Fe) was shown to maintain its complete structure, and drugs accessed its hole or absorbed on its surface. The Fourier transform infrared (FTIR) spectra are shown in Figure 3c. The spectra of Ori@MIL-53(Fe) was consistent with the spectra of MIL-53(Fe). The intensity of the absorption peak and the small changes in the position indicate some interaction between MIL-53(Fe) and Ori. The X-ray diffraction (XRD) pattern is shown in Figure 4d. The absorption peak of Ori@MIL-53(Fe) completely disappeared after taking the drug, indicating that Ori was distributed in MIL-53(Fe) in the amorphous state. Differential scanning calorimetry (DSC) spectra are shown in Figure 4e, and thermogravimetric analysis (TGA) is shown in Figure 4f. The absorption thermal peak of Ori@MIL-53(Fe) significantly changed compared with Ori, showing that the crystallinity of the drug was completely lost in MIL-53(Fe).

### 2.3. In Vitro Release Studies

**Figure 5:**
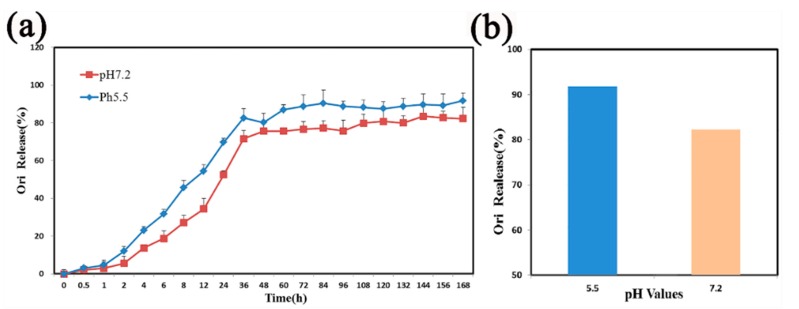
(**a**) The release curve of Ori@MIL-53(Fe) under different pH values. (**b**) The total release of Ori.

The Ori release profiles of Ori@MIL-53(Fe) was explored at two different pH values: 7.2 and 5.5. As shown in Figure 5a, the MIL-53(Fe)@Ori demonstrated a pH-responsive release processes. 

**Figure 6:**
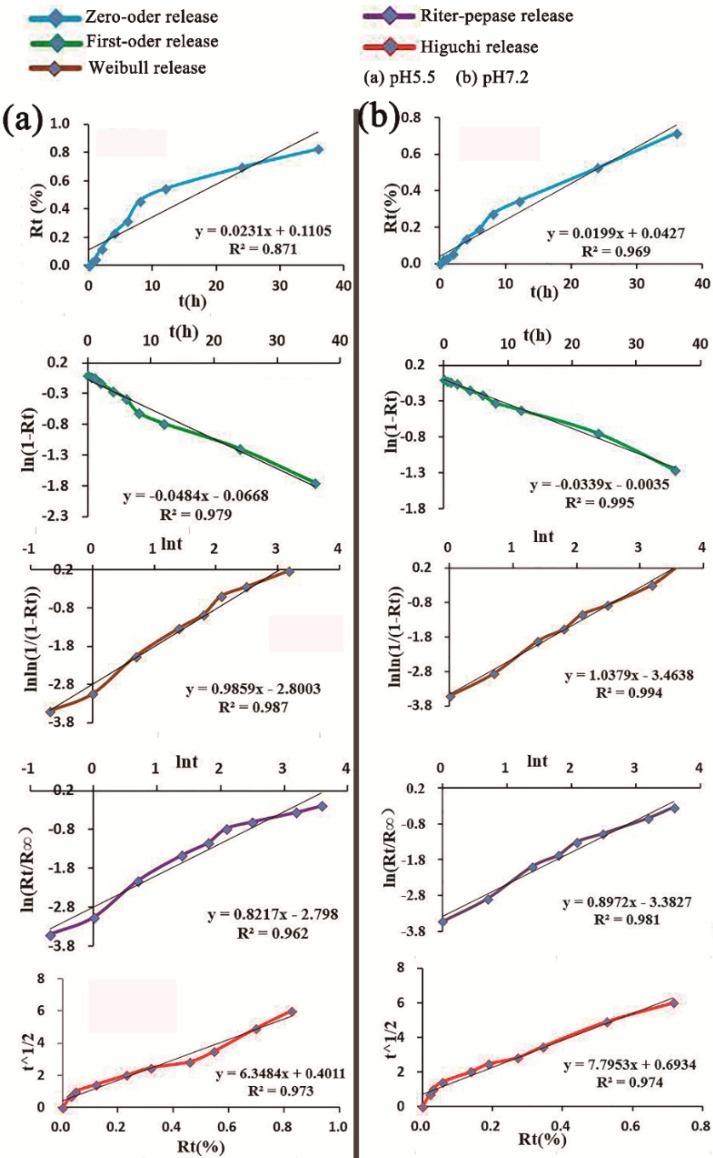
Fitting curve by different mathematical models under different pH values: (**a**) pH = 5.5 and (**b**) pH = 7.2, Rt = accumulated release rat.

At pH 7.2, 82.23% of Ori was released over 168 h. When the pH value was reduced to 5.5, 91.75% of Ori was released in 168 h (Figure 4b). The characteristic release parameters were explored by different mathematical models to explain the mechanism of drug release, and the fitting results are shown in Figure 5. At pH 5.5, the release profile of Ori@MIL-53(Fe) followed the Weilbull distribution with a regression factor of >0.987 (Figure 5a). Whereas at pH 7.2, the release profile of Ori@MIL-53(Fe) followed fist-order kinetics (ln(1−Ori)=kt+b) with regression factor of >0.995 (Figure 6b).

### 2.4. Safety Evaluation of MIL-53(Fe)

**Figure 7:**
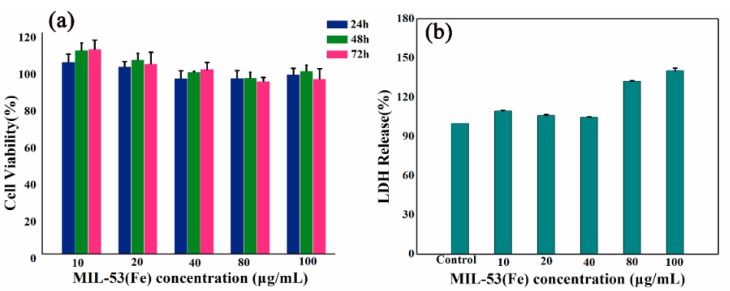
Effects of MIL-53(Fe) on cell viability and morphology. (**a**) MTT assay data were presented as mean ± SD of viability % of three independent experiments. (**b**) Evaluate HepG2 cells nuclear morphology by DAPI staining.

The results of 3-[4,5-dimethylthiazol-2-yl]-2,5 diphenyltetrazolium bromide (MTT) assays are shown in Figure 7a. There was no significant difference in the viability of HepG2 cells when MIL-53(Fe) with different concentrations were compared with vehicle controls (*p* > 0.05). In addition, the experimental results showed that MIL-53(Fe) treatment did not increase lactate dehydrogenase (LDH) release, as shown in Figure 7b. 

**Figure 8:**
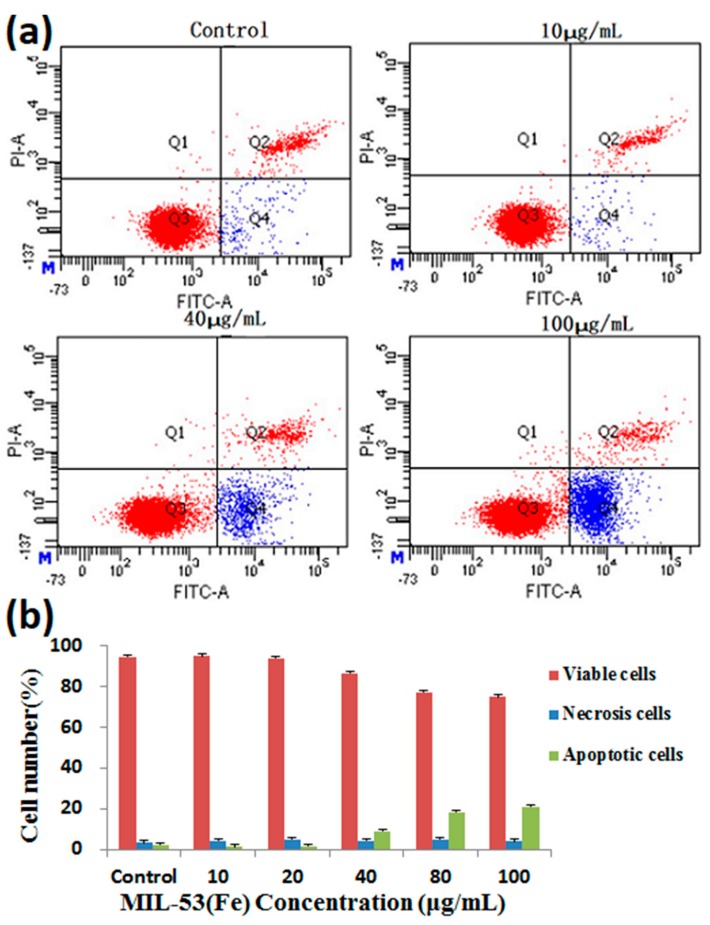
Effects of MIL-53(Fe) on apoptosis in HepG2 cells. (**a**) Flow cytometry detection of apoptosis with FITC-Annexin V/PI double staining. (**b**) The percentages of viable, early apoptosis and late apoptosis of HepG2 cells after incubation with different concentrations of MIL-53(Fe) for 24 h. The data are expressed as means ± S.D. from three independent experiments.

The percentage of viable cells, early apoptotic cells, and late apoptotic cells did not obviously change, after HepG2 cells were treated with MIL-53(Fe) for 24 h (Figure 8). The MIL-53(Fe) did not result in cells’ morphological changes and good biocompatibility of MIL-53(Fe) with HepG2 cells was observed.

### 2.5. Cytotoxic Effect of Ori@MIL-53(Fe) on HepG2 Cells

**Figure 9:**
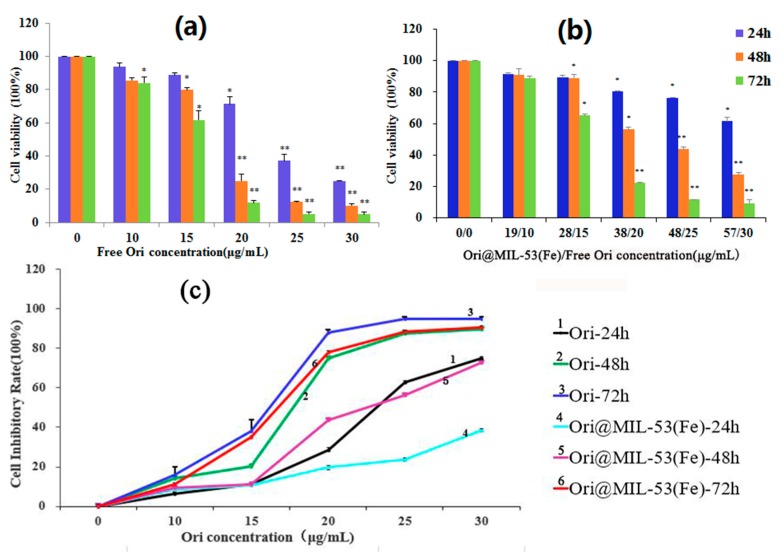
Effects of (**a**) Ori and (**b**) Ori@MIL-53(Fe) on cell viability. (**c**) HepG2 cell proliferation inhibiting rate.MTT assay date were presented as mean ± SD of viability % of three independent experiments. (* *p* < 0.05 vs. Control, ** *p* < 0.01 vs. Control).

HepG2 cells were incubated with free Ori (0, 10, 15, 20, 25, and 30 μg/mL) for 24, 48, and 72 h. As shown in Figure 9a, the treatment of HepG2 cells with Ori resulted in a significant inhibition of cell viability in both a dose- and time-dependent manner. We calculated the concentration of Ori (0, 10, 15, 20, 25, and 30 μg/mL) in Ori@MIL-53(Fe) (0, 19, 28, 38, 48, and 57 μg/mL) required to incubate HepG2 cells (Figure 9b). Ori@MIL-53(Fe) induced HepG2 cell death in a dose- and time- dependent manner, but within 48 h could not attain the same effect as free Ori in the same concentrations. This shows that Ori in Ori@MIL-53(Fe) was slowly released. However, the inhibition of Ori@MIL-53 (Fe) after 72 h reached the same level of the free drug, as shown in Figure 9c.

## 3. Discussion and Conclusions

MOFs have a wide range of compositions, crystalline structures, and crystal shapes, providing advantages over other types of materials [34]. Since the late 1990s, MOFs have been widely used in gas adsorption and separation [35] supercapacitor electrodes, catalysis, sensing [36], bio-imaging, and magnetism [37]. Some MOFs have extra-large surface areas, high-connectivity polyhedral cages, large pore volumes, high stability, nontoxicity, biocompatibility, and small sizes, making them potential excellent nanocarriers in the biomedical field [38]. These nanomedical materials can be used for various purposes, such as imaging agents for early and minimally invasive diagnosis, for specific cell and tissue (more commonly used in tumor tissue) targeting, entrapping high loadings of a drug, increased drug concentration in a local site, and creating drug-delivery formulations [39,40,41]. For example, Gordon, using an incipient wetness impregnation method, successfully loaded three model drugs (acetaminophen, progesterone, and stavudine) into MIL-53(Fe), MIL-101, and SBA-15 (20 wt %). the MIL-53(Fe) framework was slowly released for up to six days for acetaminophen in a diffusion-controlled process [31]. Motakef-Kazemi prolonged the release of ibuprofen for up to three weeks using Zn_2_(BDC)_2_(DABCO) for loading ibuprofen (30 wt %) [42]. In the present work, we synthesized a carrier material named MIL-53(Fe), which has been proven to be biocompatible, and encapsulated the anticancer drug Ori. We successfully built a drug delivery system, Ori@MIL-53(Fe), in which MIL-53(Fe) acted as the drug vehicle. This system had an efficient drug-loading capacity of 56.25 wt %, which was higher than previously reported. The reason for the increase in efficiency is that we used orthogonal design L_9_(4^3^) to optimize the drug loading process and analyze different factors affecting drug loading. This drug loading method enables MIL-53(Fe) to be used to package other drugs. 

The wide range of applications of MOFs have produced many debates among the scientific community about whether MOFs are biologically friendly in biomedical applications. Currently, the toxicity evaluation of MOFs has mostly been restricted metals and linkers individually. The literature and early results have shown that the toxicity of Mg, Fe, Zn, and Zr, as evaluated by their LD_50_, varied from a few μg/kg up to more than 1 g/kg due to the different chemical formulations [43]. The organic linkers, such as polycarboxylic or imidazolate, are not very toxic due to their high polarity and a priori easy removal under physiological conditions [44]. The flexibility with which metal clusters and organic linkers can be varied, the ability to change the physical and chemical properties of solids, and their functionalization inside and outside the surface are additional advantages of MOFs [45]. The range of cytotoxicity, host-guest interactions, hydrophobic/hydrophilic balance, biodegradability, body distribution, tissue accumulation, and excretabilty are beneficial aspects of their safety and determine whether they can be used in biological application. Before practical application of any novel MOFs, especially in a medical context, comprehensive studies of its cellular biocompatibility and nanosafety are required. In vitro toxicity tests, which are rapid, effective, inexpensive, and provide information of possible cell and nanoparticle interactions, are used to assess potential hazards of nanomaterials [46]. In this study, we used MTT assays, LDH assays, and Annexin V-FITC/PI double-staining assays to study the cell safety of MIL-53(Fe) on HepG2 cells. These assays are suitable for high-throughput screening of most new medical nanomaterials as they can establish a cytotoxicity ranking. Therefore, studying the possible toxic effects of MOFs is important.

Various new DDS can be designed according to clinical need, such as for high load capacity of different passenger molecules, tumor targeted therapy, drug release, and according to other external stimulus factors, such as pH value, temperature, light irradiation, and redox reagents [43,44,47]. From our study, we found that the release rate at pH 5.5 was faster than that at pH 7.2, which was presumed to be due to the effect of acidic environments on the conversion between the large and small hole of MIL-53(Fe), which may confirm pH-sensitive release. The portion of the drug load that was successfully incorporated within the MIL-53(Fe) framework was slowly released over as long as seven days for Ori in a diffusion-controlled process. Therefore, MIL-53(Fe) can change the pharmacokinetics of Ori, prolong circulation time, significantly enhance the antitumor effect, minimize the dosage, and reduce toxic side-effects of drugs.

In conclusion, this study reports for the first time that flexible porous MIL-53(Fe) could be used as a vector for the traditional medicine monomer Ori, with drug release at pH 5.5 showing a Weilbull distribution, and first-order kinetics drug release at pH 7.2. More than 82% of Ori in Mil-53(Fe) was released at pH values of 5.5 and 7.2. The release experiment showed that the release rate was faster in low pH environments than in higher pH environments. The MIL-53(Fe) offers many possibilities to achieve the adequately controlled release of various pharmacological molecules. Ori@MIL-53(Fe) inhibited the growth of HepaG2 cells at 28–57 μg/mL (equivalent to 15–30 μg/mL of free Ori). The suppression was both time- and dose-dependent. However, the toxicity of Ori@MIL-53(Fe) was lower than that of free Ori in equal drug concentrations, which was caused by the slow release of Ori@MIL-53(Fe). The Ori loading in MIL-53(Fe) samples proves the potential of these systems for drug delivery applications. We expect that the success of the present findings will not only provide a new approach for Ori delivery, but also pave the way for the development of new porous MOFs for practical bio-applications.

## 4. Methods

### 4.1. Synthesis and Characterization of MIL-53(Fe)

The MIL-53(Fe) was synthesized by a previously reported solvent thermal method [37]. In a typical experiment, a mixture of FeCl_3_, terephthalic acid (BDC) and *N*,*N*-dimethylformamide (DMF) with a molar ratio of 1:1:280 was mixed and sonicated for a 10 min, then placed in a Teflon lined steel autoclave, heating at 150 °C for 15 h and cooling to the temperature. Light brown powder was obtained by centrifuging using DMF as solvent and then stirring in methanol for three days. Finally, the solid powder was heated at 150 °C overnight to remove guest molecules (H_2_O, DMF, and methanol). Synthesized MIL-53(Fe) was characterized by SEM and PXRD analysis. PXRD with Cu-Kα irradiation from 5° to 50° (2θ) were used to evaluate the size, purity and crystallinity of synthesized MIL-53(Fe).

### 4.2. Stability of MIL-53(Fe) in FBS

MIL-53(Fe) was dissolved in methanol to obtain a 5 mg/L solution. FBS was diluted with PBS before testing. A 0.2 mL portion of the diluted solution of MIL-53(Fe) was added to 4.8 mL of FBS (pre-warmed to 37 °C for 30 min prior to experiments). After being incubated at 37 °C for 0, 1, 2, 4, 8, 12, 24, 48 and 72 h, observe if there is a deposit at the bottom. Zeta potential was tested with a Zetasizer (ZEN3690, Malvern Instruments Ltd., Malvern, UK).

### 4.3. Encapsulation and Evaluation of Ori@MIL-53(Fe)

The encapsulation of drugs was performed by a solvent diffusion technique. MIL-53(Fe) was added into a vial with different concentrations of Ori methanol solution, and after that, stirred for 2, 3 or 4 days at room temperature. The drug-loaded MIL-53(Fe) were then collected by centrifugation and washed with methanol three times. The concentration of Ori was determined by high performance liquid chromatography (HPLC, methanol:water = 55:45, λ = 295 nm) based on a one point external standard method. Because the Ori@MIL-53(Fe) is methanol-insoluble, the percentage of adsorbed mass of oridonin was determined by subtracting the amount of final mass from its initial mass incorporated for use, and loading capacity (LC) formula is as follows:

$$\mathrm{LC}\%=\frac{M_{0}-M_{e}}{M}\times 100\%$$

where *M*_0_ and *M_e_* are the initial amount and final amount of Ori (mg) in the system, respectively. *M* denotes the amount of Ori@MIL-53(Fe) (mg).

In addition, in order to study the physical state of the Oridonin in the porous framework, SEM, TEM, XPRD, DSC heating from 40 °C to 300 °C with a heating rate of 10.00 °C /min and FTIR were performed.

### 4.4. In Vitro Release Studies

The release of the drug oridonin from Ori@MIL-53(Fe) was estimated in PBS solution at two different pH values (pH 7.2 and pH 5.5) at 37 °C. Approximately 10 mg of Ori@MIL-53(Fe) powder wase placed in a dialysis bag (MD 34 mm) and soaked in PBS (100 mL). The drug release experiment was performed under sink conditions. At different time intervals, digestion liquor (100 μL) was extracted and filtered through a 0.45 μm polytetrafluoroethylene membrane filter. The released amount of Ori was measured by HPLC using an Agilent Zorbax SB-C18 column. Concentrations were calculated by interpolation from the calibration curves using linear regression models. The oridonin calibration curves were linear over the concentration range of 0.06 μg/mL to 84.00 μg/mL (y = 26292x − 8127, r^2^ = 0.999). The concentration was used to calculate the cumulative drug release after each time interval.

### 4.5. Cell Culture

The HepG2 cells from the hepatoma cells of human (Jenniobio Biotechnoliyg, Guangzhou, China) were cultured with the samples of the solution to evaluate the cytotoxicity. They were cultured with Dulbecco’s Modified Eagle Medium (DMEM) supplemented with 10% FBS and 1% penicillin−streptomycin solution, followed by incubation in a humidified atmosphere of 5% CO_2_ at 37 °C. The culture medium was refreshed every 2 or 3 days.

### 4.6. Cytotoxicity Assay

The cytotoxicity of Ori@MIL-53(Fe) was evaluated by a MTT assay of cellular viability on the hepatocellular carcinoma HepG2 cells from human. HepG2 cells were seeded in 96-well plates at a density of 3 × 10^3^ cells per well. After the cell attached to the wall, the solutions with different concentrations of MIL-53(Fe), Ori@MIL-53(Fe) and free Ori were added into the well and continued to be cultured for 24 h, 48 h and 72 h. After the prescribed time, the MTT solution with a concentration of 5 mg/mL was added to each well and incubated for 4 h at 37 °C. Culture supernatant was removed from all the wells, dimethyl sulfoxide (DMSO, 150 μL) was added to each well, and the plate was shaken for 10 min. Absorbance of the formazan solution was read at 570 nm in a microplate reader. The results were expressed as the percentage of cell viability. All tests were repeated three times.

### 4.7. LDH Assay 

Lactate dehydrogenase (LDH), which is present mainly in the cytoplasm and exists in the extracellular medium, is used to investigate cell membrane integrity damage. LDH leakage is considered as a sign of cell membrane disruption [48]. For the measurement of LDH, HepG2 cells were seeded into 96-well plates overnight and then treated with various concentrations of MIL-53(Fe) for 48 h. After 48 h treatment, the supernatant was collected to determined LDH activity with a commercial kit. All of the experimental tests were performed three times.

### 4.8. Annexin V/PI Double-Staining Assay

Apoptotic cells were quantified using an Annexin V-FITC detection kit and analyzed by flow cytometry. In this process, HepG2 cells were seeded in 6-well plates at a density of 3.5 × 10^5^ cells/well and treated with different concentrations of MIL-53(Fe) for 24 h at 37 °C. The cells were washed with PBS and resuspended in 295 µL binding buffer. Annexin V-FITC (5 µL) and PI (10 µL) were added, and the mixture was incubated for 20 min at 37 °C in the dark. Finally, apoptotic cells were immediately analyzed by flow cytometry.
